# Pregnancy Outcomes in Women of Advanced Maternal Age: a Retrospective Cohort Study from China

**DOI:** 10.1038/s41598-018-29889-3

**Published:** 2018-08-16

**Authors:** Dan Shan, Pei-Yuan Qiu, Yu-Xia Wu, Qian Chen, Ai-Lin Li, Sivakumar Ramadoss, Ran-Ran Wang, Ya-Yi Hu

**Affiliations:** 1 Department of Gynaecology and Obstetrics, West China Second University Hospital, Sichuan University, Chengdu, China; 2 Key Laboratory of Birth Defects and Related Diseases of Women and Children, Sichuan University, Ministry of Education, Chengdu, China; 3 West China School of Public Health, Sichuan University, Chengdu, Sichuan China; 4 Department of Ophthalmology, Chengde Medical University, Chengde, Hebei China; 5 Department of Obstetrics and Gynaecology and Department of Molecular and Medical Pharmacology David Geffen School of Medicine at University of California at Los Angeles; Jonsson Comprehensive Cancer Centre, Los Angeles, CA USA; 6 West China School of Pharmacy, Sichuan University, Chengdu, Sichuan China

## Abstract

This retrospective cohort study attempts to investigate pregnancy complications and adverse pregnancy outcomes in women of advanced maternal age (AMA). Data were extracted from electronic medical records system at West China Second University Hospital of Sichuan University from January 2013 to July 2016. The study cohort consisted 8 subgroups of women on 4 different age levels (20–29 years, 30–34 years, 35–39 years and ≥40 years) and 2 different parities (primiparity and multiparity). In the study period, 38811 women gave birth at our hospital, a randomized block was used to include 2800 women of singleton pregnancy >28 gestational weeks, with 350 patients in each subgroup. Maternal complications and fetal outcomes were collected and defined according to relevant guidelines. Confounding factors representing maternal demographic characteristics were identified from previous studies and analysed in multivariate analysis. There was an increasing trend for the risks of adverse pregnancy outcomes with increasing age, especially in AMA groups. Our study showed that AMA, primiparity, maternal overweight or obesity, lower educational level and residence in rural area increased pregnancy complications and adverse fetal outcomes. Increased professional care as well as public concern is warranted.

## Introduction

The past several decades have witnessed a remarkable shift in the demographic changes of childbearing age worldwide. In the United States, the birth rate for women aged 35–39 was 51.0 births per 1,000 women in 2014, up 3% from 2013 (49.3‰). But the birth rate in women of 20–24 age group declined from 80.7‰ in 2013 to 79.0‰ in 2014, considering this rate was as high as 115.1‰ in 1980^1^. In Japan, the rate of birth to women aged 35 above elevated from 8.6% in 1990 to 25.9% in 2012^2^. Similar trends have been found in other developed countries^3–6^. In China, the shifting demographic change of delaying child birth is as well on the trend. The birth rate in women aged 35 to 39 increased from 8.65‰ in 2004 to 17.04‰ in 2014, and in the 40–44 age group, the rate raised from 1.77‰ to 3.96‰. In contrast to that, birth rate in women of 25–29 age group decreased from 102.44‰ to 93.62‰^7^. The ratio for advanced pregnancies was about 31% of the total pregnancies in 2016^8^.

It is an undeniable fact that fecundity decreases with age. But this anti-natural trend of delaying child birth occurred for several reasons. Chinese government has relaxed its more than three-decade-old family planning policy and enacted the universal two-child policy in 2015 to address the country’s aging issue. That with no doubt will lead this trend steadily on the rise.

The association of advanced maternal age (AMA) and increased risk of congenital anomaly, spontaneous abortion, perinatal mortality and maternal complications have been reported in many studies in developed countries^3–6,9–11^. While majority of these studies give impressive findings of adverse connection of age and pregnancy outcome, some studies have yielded inconsistent conclusions^12,13^. Whether the disadvantages originated in aged pregnancies could be compensated by the advantages derived from high social economic status of older mothers and medical advances still need to be explored. In addition, there is no consensus among researchers regarding the precise maternal age as to when the association of adverse pregnancy outcome and increasing age becomes clinically important^3,14–16^.

The term “elderly primigravida” was first used in 1950^17^. Some studies revealed that primiparity was more likely to be related to adverse maternal outcomes^10,12^. But others found a united effect of both age and parity^11,18^. Like other fast-developing countries, obesity is an important public health issue in China. Studies found that obesity was more common in aging women and was associated with several adverse pregnancy outcomes such as preeclampsia, gestational diabetes mellitus (GDM) and stillbirths^19–21^. In some rural areas in China, patients are at low educational level. Lack of antenatal care and ignorance of pregnancy complication related symptoms may pose great threat to maternal and fetal health. With the advent of “universal two-child policy”, their strong fertility desire of older women may make the existing problems even worse in this population.

Even though, the age associated complications and on pregnancy outcome was studied in developed countries, there were only few studies in Chinese population. China is a developing country with unique socioeconomic characteristics of pregnancies. The impact of demographic factors on pregnancy outcome in Chinese population is also very limited. Therefore, here we attempted to investigate the association between advanced maternal age and risks of pregnancy complications and adverse pregnancy outcomes in China. We also explored other demographic factors which could affect pregnancy outcomes.

## Results

**Figure 1:**
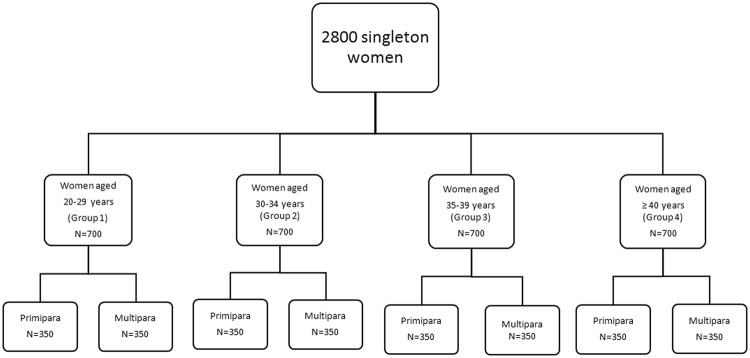
Study flow chart.

A total of 2800 patients were included in our study (Fig. 1). The demographic characteristics are reported by categories of maternal age in Table 1. There were 27 patients aged 45 and above in group 4, 10 of them were primiparous mothers. We found that increasing maternal age was associated with increasing gravidity times and rates of assisted reproductive technology (ART) pregnancies. More women of AMA groups were likely to be categorized as overweight or obese. Especially in women aged 40 and above. Women in the referent group were better educated than women in the AMA groups.

**Table 1:** Characteristics of the study population by maternal age groups.

Characteristic	Maternal age (years)			
	Group1 20–29 (N = 700)	Group2 30–34 (N = 700)	Group3 35–39 (N = 700)	Group4 ≥40 (N = 700)
Age (y), mean (SD)	26.05 ± 2.48	32.31 ± 1.28	36.70 ± 1.37	41.14 ± 1.47
Gestational weeks (w), mean (SD)	38.55 ± 2.09	38.85 ± 1.64	38.55 ± 1.84	38.32 ± 2.01
Baseline BMI (kg/m^2^), mean (SD)				
Underweight, n (%)	194 (27.7)	109 (15.6)	58 (8.3)	55 (7.9)
Normal, n (%)	448 (64.0)	529 (75.5)	533 (76.1)	512 (73.1)
Overweight, n (%)	53 (7.6)	53 (7.6)	76 (10.9)	103 (14.7)
Obese, n (%)	5 (0.7)	9 (1.3)	33 (4.7)	30 (4.3)
Gravidity, n (%)				
1	207 (29.6)	185 (26.4)	101 (14.4)	86 (12.3)
2	237 (33.8)	209 (29.9)	158 (22.6)	129 (18.4)
≥3	256 (36.6)	306 (43.7)	441 (63.0)	485 (69.3)
Insurance, n (%**)**	346 (49.4)	540 (77.1)	406 (58.0)	417 (59.6)
Education, n (%)				
Low, n (%)	52 (7.4)	34 (4.9)	83 (11.9)	69 (9.9)
Middle, n (%)	203 (29.0)	163 (23.3)	324 (46.3)	380 (54.3)
High, n (%)	445 (63.6)	503 (71.8)	293 (41.9)	251 (35.9)
Area of residence, n (%)				
Metropolitan	319 (45.5)	546 (78.0)	513 (73.3)	506 (72.3)
Remote	179 (25.6)	99 (14.1)	110 (15.7)	86 (12.3)
Rural	202 (28.9)	55 (7.9)	77 (11.0)	108 (15.4)
ART, n (%)	7 (1.0)	20 (2.9)	86 (12.3)	113 (16.1)

Note: BMI: body mass index; ART: assisted reproductive techniques; SD: standard deviation.

### Association between AMA and adverse pregnancy outcomes

In the multivariate analysis, the associations between maternal age and adverse pregnancy outcomes were identified. Table 2 indicated an increasing trend for the risks of adverse pregnancy outcomes with increasing age, especially in AMA groups (group 3 and 4). The sharp contrast for the risk of ART pregnancies in AMA groups confirmed the fertility decline in women older than 35. In comparison with the referent group, the adjusted odds ratio for ART pregnancy were 3.2, 17.1 and 25.0 in group 2, 3 and 4 respectively. The risk for preeclampsia of patients in group 4 was almost 7 times of that in group 1, but in group 3 it were only 4 times. Risk for GDM (lifestyle intervention) was almost 4 times in group 4 of that in group 1, however in women of group 3, it was only 2.3 times. Women in group 4 also had higher incidences of selective caesarean section (CS) in comparison with group 3 and group 2.

**Table 2:** Crude and adjusted relative risks of the association between maternal age and adverse pregnancy outcomes.

	Maternal age 30–34 years		Maternal age 35–39 years		Maternal age ≥40 years	
	Crude OR [95% CI]	Adjusted OR^a^ [95% CI]	Crude OR [95% CI]	Adjusted OR^a^ [95% CI]	Crude OR [95% CI]	Adjusted OR^a^ [95% CI]
**Maternal Outcomes**						
Selective CS	1.53 [1.23 1.915]^b^	1.45 [1.14 1.83]^b^	2.48 [2.00 3.09]^b^	2.13 [1.67 2.71]^b^	4.21 [3.37 5.27]^b^	3.55 [2.75 4.58]^b^
Emergency CS	1.07 [0.83 1.37]	1.07 [0.82 1.40]	0.95 [0.74 1.23]	0.86 [0.65 1.14]	0.98 [0.77 1.27]	0.84 [0.63 1.13]
ART	2.91 [1.22 6.93]^b^	3.24 [1.33 7.90]^b^	13.87[6.37 30.19]^b^	17.10[7.59 38.51]^b^	19.06[8.81 41.21]^b^	24.95[11.04 56.39]^b^
Preeclampsia	1.68 [0.73 3.87]	2.32 [0.98 5.52]	3.80 [1.80 8.00]^b^	3.97 [1.81 8.74]^b^	7.46 [3.68 15.14]^b^	7.33 [3.43 15.68]^b^
Severe preeclampsia	1.51 [0.67 3.39]	2.37 [1.00 5.57]	2.56 [1.22 5.36]^b^	3.18 [1.42 7.10]^b^	2.98 [1.44 6.17]^b^	3.35 [1.51 7.45]^b^
Early onset preeclampsia	1.85 [0.68 5.02]	2.22 [0.77 6.41]	2.88 [1.13 7.35]^b^	2.63 [0.95 7.26]	2.53 [0.98 6.57]	2.03 [0.72 5.73]
Gestational Hypertension	1.20 [0.37 3.96]	1.15 [0.34 3.94]	5.15 [1.96 13.53]^b^	4.75 [1.70 13.29]^b^	2.63 [0.93 7.42]	2.52 [0.83 7.66]
GDM	1.30 [0.98 1.72]	1.39 [1.00 1.87]	2.78 [2.15 3.61]^b^	2.51 [1.89 3.33]^b^	3.26 [2.52 4.21]^b^	2.90 [2.16 3.88]^b^
GDM (lifestyle intervention)	1.15 [0.83 1.58]	1.20 [0.86 1.69]	2.50 [1.87 3.35]^b^	2.32 [1.69 3.19]^b^	4.10 [3.10 5.43]^b^	3.81 [2.77 5.24]^b^
GDM (on insulin)	1.61 [0.99 2.62]	1.77 [1.05 2.98]	2.46 [1.56 3.88]^b^	2.12 [1.27 3.52]^b^	0.67 [0.37 1.21]	0.55 [0.29 1.04]
ICP	0.64 [0.40 1.01]	0.84 [0.51 1.36]	0.87 [0.57 1.33]	1.14 [0.71 1.84]	0.83 [0.54 1.27]	1.13 [0.69 1.87]
Placental abruption	1.11 [0.45 2.76]	0.94 [0.36 2.46]	1.23 [0.51 2.98]	1.23 [0.46 3.30]	0.78 [0.29 2.09]	0.79 [0.26 2.41]
Placenta Praevia and Vasa Praevia	1.28 [0.87 1.87]	1.49 [0.99 2.25]	1.32 [0.90 1.93]	1.14 [0.75 1.73]	2.05 [1.44 2.92]^b^	1.60 [1.06 2.41]^b^
PPH	1.17 [0.72 1.89]	1.38 [0.82 2.33]	0.74 [0.43 1.27]	0.65 [0.36 1.17]	1.30 [0.81 2.09]	1.07 [0.62 1.85]
Abnormality of fetal presentation (non-cephalic)	1.16 [0.72 1.85]	1.18 [0.71 1.94]	1.54 [0.98 2.41]	1.34 [0.81 2.20]	1.57 [1.01 2.45]^b^	1.31 [0.78 2.20]
**Fetal outcomes**						
Preterm birth (<37 weeks)	0.55 [0.39 0.77]^b^	0.65 [0.45 0.94]^b^	0.71 [0.51 0.97]^b^	0.70 [0.49 0.99]^b^	0.84 [0.62 1.14]	0.80 [0.56 1.15]
Low birthweight (<2500 g)	0.63 [0.40 0.98]^b^	0.76 [0.47 1.23]	0.83 [0.55 1.27]	0.81 [0.50 1.30]	1.01 [0.67 1.51]	0.93 [0.58 1.49]
Macrosomia (>4000 g)	1.45 [0.91 2.32]	1.38 [0.84 2.29]	1.10 [0.67 1.81]	0.98 [0.57 1.70]	0.94 [0.56 1.57]	0.81 [0.45 1.46]
IUGR	0.20 [0.04 0.91]^b^	0.21[0.04 1.02]	1.00 [0.41 2.41]	0.71 [0.25 1.98]	0.90 [0.37 2.24]	0.58 [0.20 1.73]
Apgar score < 7 at 5 minute	0.67 [0.11 4.00]	1.28 [0.18 8.88]	0.67 [0.11 4.00]	1.41 [0.20 10.13]	1.34 [0.30 6.02]	2.93 [0.49 17.70]
NICU admission	0.54 [0.34 0.86]^b^	0.62 [0.38 1.02]	0.78 [0.51 1.18]	0.72 [0.45 1.15]	0.86 [0.57 1.30]	0.73 [0.45 1.18]
Respiratory complications	2.91 [1.22 6.93]^b^	2.45 [0.99 6.06]	2.61 [1.08 6.30]^b^	2.05 [0.80 5.25]	2.62 [1.09 6.33]^b^	1.86 [0.70 4.92]

Note: CI: confidence interval; OR = odds ratio; ART: assisted reproductive techniques; GDM: gestational diabetes mellitus; ICP: Intrahepatic cholestasis of pregnancy; PPH: postpartum haemorrhage; IUGR: Intrauterine growth restriction; NICU: neonatal intensive care unit.

^a^Adjusted for gravidity, parity, maternal baseline BMI, conceiving method, insurance, education, and residence. In the comparison of “conceived by ART”, the aforementioned confounding factors were considered except for conceiving method. ^b^Denotes significance with a CI that does not cross 1

Maternal age 20–29 as the referent group. In the analysis of fetal outcomes, 2796 patients were analysed excluding pregnancies ending in stillbirth.

Patients in group 2 and 3 had lower risk for preterm birth. We included preeclampsia and GDM as confounding factors into the analysis in another model. For the reason that these two complications were more common in our AMA patients compared with other pregnancy complications. These two factors were also reported to increase the risks for adverse perinatal outcomes^22–24^. After adding these two factors, we found that advanced maternal age was a protective factor for preterm birth and neonatal intensive care unit (NICU) admission (For preterm birth, adjusted OR 0.61, 95% CI 0.42–0.88 in group 2; adjusted OR 0.60, 95% CI 0.42–0.87 in group 3; adjusted OR 0.62, 95% CI 0.42–0.91 in group 4. For NICU admission, adjusted OR 0.57, 95% CI 0.34–0.94 in group 2; adjusted OR 0.60, 95% CI 0.37–0.98 in group 3; adjusted OR 0.53, 95% CI 0.31–0.88 in group 4). There were four stillbirths, all of them were in women aged 40 and above, 3 of them were in primiparous mothers. Two patients were below 30 gestational weeks in mothers with pernicious placenta praevia and HELLP syndrome (haemolysis, elevated Liver enzymes, and low platelet count). Two patients were between 32 to 33 gestational weeks. One of them was in a multiparous patient diagnosed with severe intrahepatic cholestasis of pregnancy and had poor compliance to prenatal care advices. The other one suffered severe anoxia in labour.

### Association between demographic factors and adverse pregnancy outcomes

To investigate the influence of maternal demographic characteristics, our multivariate analysis included potential confounding factors from previous studies (the influence of these factors can be found as Supplementary Tables S1 and S2). Few patients in our study could be categorized as overweight or obese according to the World Health Organization (WHO) definitions. Considering this, we combined the overweight group and obese group together in our analysis. Higher maternal baseline body mass index (BMI) was associated with increased risks for emergency CS, GDM (on insulin), and low birthweight. It also increased the risks for preeclampsia, brought the onset time earlier and aggravated the severity. Lower maternal BMI was associated with higher risks of low birthweight (adjusted OR 1.65, 95% CI 1.09–2.49) and placenta previa (adjusted OR 1.50, 95% CI 1.05–2.14). Compared with multiparous women, primiparous women had higher incidence for ART pregnancies, GDM (lifestyle intervention) and placenta previa. Conceived by ART increased the selective CS risk. Patients of more gravidity times had elevated rates for placenta previa (adjusted OR 1.26, 95% CI 1.16–1.36), postpartum haemorrhage (PPH) (adjusted OR 1.15, 95% CI 1.03–1.29) and abnormal fetal presentation (adjusted OR 1.14, 95% CI 1.03–1.27).

Lower educational level increased risks for many maternal complications, including preeclampsia, early onset preeclampsia, severe preeclampsia, GDM (on insulin), placenta previa and PPH. Risks for adverse perinatal outcomes such as preterm birth, low birthweight and NICU admission were elevated in mothers of low educational level as well. Compared with patients living in metropolitan, residents of rural area had higher incidence of preeclampsia, preterm birth, low apgar score and NICU admission. Patients with insurance were less likely to have preeclampsia and GDM.

### Associations between parity with adverse pregnancy outcomes according to different age levels

**Figure 2:**
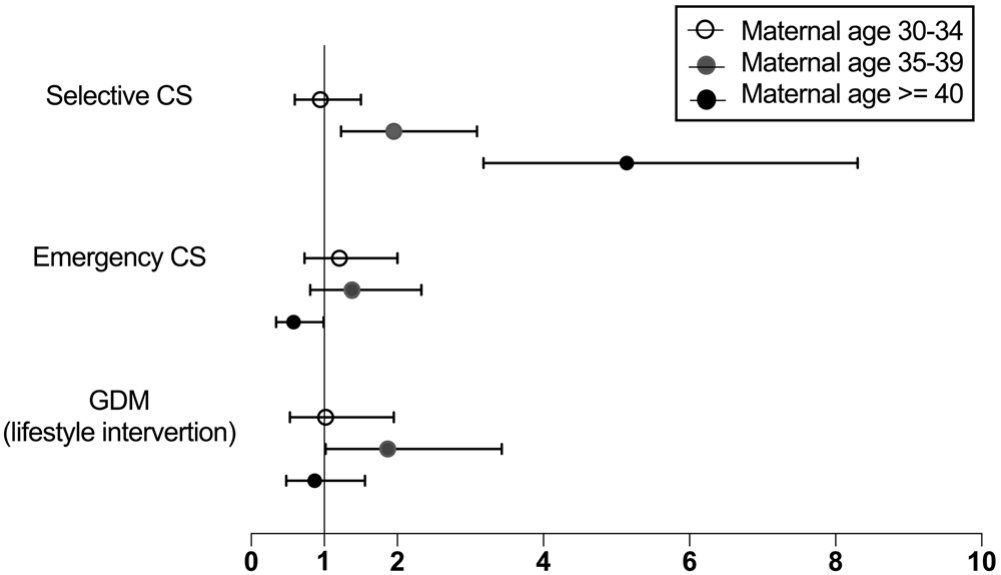
Risks of caesarean sections and gestational diabetes in primiparous women compared with multiparous women in different age levels (30–34 years, 35–39 years and ≥40 years). Note: Forest plot of odds ratios with 95% CIs in multivariate logistic regression analysis, after adjustment for gravidity, maternal baseline BMI, conceiving method, insurance, education, and residence. Multiparous women as the referent group. CS: caesarean section; GDM: gestational diabetes mellitus.

Figure 2 presented the different effect of parity on maternal outcomes in different age levels. Primiparity increased the selective CS risks with increasing maternal age, especially in AMA groups (adjusted OR 1.95, 95% CI 1.23–3.09 in group 3; adjusted OR 5.14, 95% CI 3.18–8.30 in group 4). However, for emergency CS, there was a reduced risk in primiparous patients aged 40 years and above (adjusted OR 0.58, 95% CI 0.34–0.99 in group 4). As an aggravating factor for GDM (lifestyle intervention), primiparity increased this risk in patients of group 3, while it had no effect on in older or younger counterparts (adjusted OR 1.87, 95% CI 1.02–3.43 in group 3).

## Discussion

This study attempts to shed more light on the hypotheses surrounding older mothers, primiparae and multiparae of the same number on different age levels were included. Our research revealed that, risks for selective CS, GDM and hypertensive disorders were elevated in older pregnant women, there was a trend of increasing risks with increasing maternal age. AMA did not increase risks of PPH, preterm birth, low Apgar scores or NICU admission. Primiparity was related with higher rates of GDM (lifestyle intervention) and placenta previa. Being overweight or obese is the aggravating factor for hypertensive disorders, GDM (on insulin), emergency CS and low birthweight. Low educational level and residence in rural area accounted for the elevated risks of several adverse pregnancy outcomes such as hypertensive disorders, preterm birth and neonatal NICU admission.

Direct biological changes and environmental impacts accumulated with increasing age. Increased susceptibility to pregnancy-related complications in AMA mothers may partly stem from inadequate maternal cardiovascular adaptations during pregnancy^25,26^. Due to these inadequate adaptations, the dramatic hemodynamic changes for supporting the fetus could not be guaranteed. Of note, animal studies also showed in aged dams, reduced nitric oxide bioavailability can lead to altered endothelium function and loss of cardiovascular compliance, and resulted in a more constrictive vasculature in both uterine and systemic circulation^26,27^. In addition, the invading capacity of the trophoblast cells into the underlying decidua is constrained because of impairment of decidua reaction as well as changes in microvillus architecture^26^. Furthermore, ischemic placenta could trigger more oxidative stress reaction, thus lead to increased syncytiotrophoblast apoptosis and more immunological responses which lead to higher risks of pregnancy complications^28,29^.

Overall 4.3% patients in our study were diagnosed with preeclampsia, but the rate was increased to 6.8% in women of AMA groups. In group 1 and group 2, the rate was 1.3% and 2.1% respectively. This finding revealed the dramatic increasing trend of the risk of preeclampsia with maternal age. Our findings were in consistent with 2 other studies proposed in China, but not other studies in USA, UK, Germany and Israel^4,9,10,30–32^. In Khalil’s study, which included patient’s histories of disease and family histories into the multivariate analysis, the risk of preeclampsia was revealed to be only 1.5 fold^4^. In other 3 studies, the risk was not higher than 2 fold^9,10,32^. Differences in racial origin and analysis method may partly contribute, living habits like smoking and alcohol consumption may lead to this difference as well. Only 6 patients in our study have regular smoking habit, which is very prevalent among women in western countries. In addition, an epidemiologic study conducted in 3 different medium-sized cities in China revealed low prevalence of preeclampsia^33^. Except for ethnicity difference, the authors contributed the low prevalence to lower average BMI and healthier lifestyle. In our analysis, this low prevalence of preeclampsia in the referent group may make the influence of aging more prominent.

It is noteworthy that our analysis revealed being overweight or obese elevated risks of preeclampsia. The incidence for the early onset type and severe type were increased to 3.7 and 3.5 times respectively comparing patients of normal BMI. 15.6% patients were categorized as overweight or obese in group 3, this ratio was 19.0% in group 4. Higher BMI in older mothers might make the existing oxidative damage reaction even worse^24^. Lower educational level and living in rural area acted as aggravating factors for both the severe type and early onset type of preeclampsia. Studies in other developing countries found higher risks for preeclampsia in residents of rural area as well, preeclampsia was the leading cause for maternal death of rural places in some countries^34,35^. Lack of antenatal care, no perception of hazardous condition and long-time exposure to hypertension were the underlying reasons in this population, which lead to the higher incidence of the severe type and early-onset type rather than the normal type.

There was a 2–3 fold risk for AMA mothers to be diagnosed with GDM in our analysis. This ratio was also revealed by other studies^5,9^. This increase might stem from the reduction in insulin sensitivity and more dysfunctional lipid profile metabolism with aging^36,37^. By stratifying patients at different age levels, we found primiarity significantly increased this rate in group 3. Some studies suggested that adaptations of the haemodynamic system during the first pregnancy could permanently reconstruct the uterine arteries, decrease vascular resistance thus reduce constrictive cytokines and lead to lower risks of maternal complications in next gestation^38^. In younger patients, maternal cardiovascular system had good adaptability. The reconstruction might be unnecessary for there were no difference between primipara and multipara. Our finding confirmed the cutting point at 35 years as the definition of “elderly primigravida”^12,17^. In mothers ≥40 years, healthy maternal lifestyle might contribute to compensate. Maternal neuroendocrine and inflammatory processes are also thought to be influencing factors for pregnancy outcomes^39,40^. Better lifestyle might contribute to guarantee a healthy internal environment thus reduced the risks for maternal complications, especially for GDM, only because GDM was easily controlled by self-regulated behaviour compared to other complications. However, as for higher maternal baseline BMI, we found that more insulin intervention were needed. Enhanced monitoring of blood glucose levels and strict weight control plan should be implemented. Association of low educational level and GDM yielded the need for the improvement of public awareness.

Demand for selective CS increased with increasing maternal age. There were 2.1 and 3.5 fold of selective CS in group 3 and group 4 in comparison with group 1. In AMA mothers, higher rates of pregnancy complications maybe one of the proposed causes. Considering the reduced number of oxytocin receptors and the incompetent capability of contraction of the aging myometrium, CS might be an easy choice for doctors as well. However, maternal request is another important and undeniable factor. Studies in Asian, European and American countries reported the CS rate in women older than 35 varied from 53.3% to 91.8%^6,9,15,18^. A retrospective study performed in Boston found women aged ≥40 were more likely to have elective CS without medical indications^41^. A study in Beijing included 15 hospitals reported that CS rate was 66.3% in women of 35–39 years and 74.8% in women ≥40 years^31^. Similar to the situation in these studies, some CS were performed following strong maternal request in our hospital. This tendency was further confirmed by our finding of the trend that the influence of primiparity on selective CS was elevated with increasing age. Highest rate of selective CS and lowest rate of emergency CS were both in primiparous women ≥40 years. These findings reflected maternal and obstetrician preferences of a low threshold for risk avoidance. In addition, the increased emergency CS rate caused by higher incidence of having difficulties during labour in obese and overweight mothers indicated the importance of weight control in these patients on the other hand.

Unlike the previous reports conducted in America, UK and Nigeria, AMA was not associated with PPH risk in our study^42–44^. Another Chinese study revealed a decreasing odds of PPH with increasing age as well^45^. In recent years, Chinese government has taken action to control the PPH rate^7,8^. Our hospital is the organizer agency for Chinese PPH guideline. There were systematic special trainings in doctors, anaesthetists and midwives for the prevention of PPH. Patients with high risk factors for PPH, especially the AMA mothers, were well educated in pregnancy school. We had monthly meetings discussing PPH cases, which helped to improve the prevention of similar cases in future.

As with other studies, we did not find the association between AMA and adverse fetal outcomes. After including preeclampsia and GDM into the multivariate analysis, we found lower risks of preterm birth and neonatal NICU admission in older mothers. Similar findings from European and American countries revealed decreased neonatal adverse outcomes in AMA mothers as well, some even found an inverse relationship^4,9,13^. Self-regulated maternal behaviour might be one reason. Survivorship of healthier embryos or fetus in AMA mothers might be the second reason. After suffering from infertility and miscarriage^11,18^. Natural selection made only healthier embryos can survive and avoid adverse pregnancy outcomes in AMA women^11,18^. Third, women of older age experienced less psychosocial stress compared to young women, who was in the process of social transformation thus might be under huge pressure and experience mood swings. Psychosocial stress has been implicated as a risk factor for preterm birth^46^. Better coping mechanism and socioeconomic support led to better neonatal outcomes. Of note, the influence of lower educational level and residence also influenced the fetal outcomes. This finding refleceted the importance of education, which had been considered as a very potent influencing factor for perinatal outcomes^47,48^. Like AMA mothers, similar underlying mechanisms might be the reasons for better educated mothers. Because they usually had privileges in the labour market which guaranteed better socioeconomic support. They usually behaved well with self-regulation. The increased risks for adverse perinatal outcomes in patients from rural area confirmed the urgent needs for improving the awareness of antenatal care in small cities and villages.

However, all of the 4 stillbirths in our study were in mothers aged 40 and above. The total stillbirth rate in China in the year 2015 was 5.4‰, in urban population the rate was 3.3‰^8^. Our stillbirth rate of 5.7‰ was slightly higher than the overall rate in China but in consistent with other studies including women aged 40 and above, in which the stillbirth rate ranged from 5‰ to 55‰^11,15,49,50^. 3 stillbirths were in mothers with severe pregnancy complications and one happened during labour. A Scottish study revealed that women aged 40 and above had greater than a 2 fold increased risk of delivery related perinatal death^51^. This evidence verified the risk-averse choice of selective CS instead of vaginal delivery in doctors on the other hand. Comprehensive prenatal care and strict weight control should be implemented. Intensive monitoring and adequate preparation are needed during labour, especially in patients aged ≥40 years.

In conclusion, our findings of the associations between maternal age, baseline BMI, primiparity, educational level and residence with adverse pregnancy outcomes represented the need to control these modifiable risk factors from a public health perspective. To challenge the continuing rapid increase in maternal age in China, increased professional care as well as public concern is warranted. Preventative strategies to halt the increasing trend of obesity and popularization of public education were also needed. In rural areas, improvement in public awareness of pregnancy related complications and better implementation of antenatal care service are also necessary. Moreover, antenatal care in AMA mothers should emphasize on the prevention of GDM and hypertensive disorders.

This study addresses a significant gap in the literature on the outcomes of AMA mothers in Southwest China. Equal number of primiparous and multiparous women of different age levels were included by a computer generated randomized block. Patients deriving from a single medical centre ascertained the same diagnostic criteria, treatment, and prenatal monitoring. This study has limitations that should be noted. Potential confounding factors were identified from previous studies, medical records rather than direct patient interview were used. There are possible underlying confounding factors that we could not take into account. This might have influence on the result. The possibility of selection bias could not be excluded for the reason that study was performed in a tertiary referral centre. The results from this study may not be generalizable to women of very advanced maternal age (≥45 years) or women of multiple pregnancies.

## Methods

### Populations and settings

We performed a retrospective cohort study at West China Second University Hospital of Sichuan University in Chengdu, China. The Ethics Committee of West China Second University Hospital of Sichuan University approved our study. All of the health care procedures in our hospital were in accordance to the approved guidelines and regulations. Medical records of women with a singleton fetus delivered above 28 gestational weeks from January 2013 to July 2016 were obtained from the electronic medical records system in our hospital. This system has been used for more than 10 years and provided valid and accurate data for several epidemiological studies, including case control studies, cohort studies and nationwide surveys from our hospital. In this system, maternal demographic characteristics, treatment procedures and pregnancy outcomes were all included. The International Statistical Classification of Diseases and Related Health Problems 10th Revision (ICD-10) were used in this system. Informed consent was obtained from all participants. Women with critical illness before pregnancy and those gave up pregnancies because of serious congenital fetal abnormalities were excluded. All patients were followed up till the twelfth week day after delivery, data form the postpartum clinic was also analysed.

The total number of pregnancies in our hospital were 38811 in the study period. 15712 pregnant women were aged ≤29 years, 13305 women aged between 30–34 years, 8084 women aged between 35–39 years and 1710 women aged ≥40 years. Due to the large amount of patients, we could not retrieve all these data. The study cohort consisted of women in 4 different age groups: group 1(referent group, maternal age of 20–29 years), group 2 (maternal age of 30–34 years), group 3 (maternal age 35–39 years) and group 4(maternal age ≥40 years). Group 3 and 4 compromised the AMA groups. Each age group consisted of 2 subgroups with equal number of primiparities or multiparities. Considering the number of primiparous women aged 40 years and above was small, we first confirmed the number of this subgroup and located 404 patients, after excluding patients with incomplete data, multiple pregnancies and gestational weeks <28, 350 patients were included. Then a computer generated randomized block was used to screen patients in other 7 subgroups. This randomized block was based on the unique admission number for each medical records in our hospital.

### Data collection and variables definitions

Data on maternal demographics, obstetrics and perinatal outcomes were collected for all patients. Previous studies focusing on AMA issues and pregnancy complications indicated the potential influence of maternal baseline BMI, parity, conceiving method, educational level, residence, insurance status and gravidity times on pregnancy outcomes^3–6,9,15,18,19,45^. We included these potential confounding factors into our multivariate logistic regression model. The maternal outcomes include pregnancy complications and CS rate. Pregnancy complications such as preeclampsia, severe preeclampsia, early onset preeclampsia, gestational hypertension, gestational diabetes mellitus on lifestyle intervention or insulin, intrahepatic cholestasis of pregnancy (ICP), placenta praevia or vasa praevia, placental abruption, PPH, and ART pregnancies were recorded. The fetal outcomes studied were stillbirth, preterm birth, low birth weight (<2500 g), macrosomia (≥4000 g), intrauterine growth restriction (IUGR), Apgar scores, NICU admission rate and complications of respiratory tract of the newborns.

Gestational age was calculated with reference to the first trimester ultrasound scan and data from the last menstrual period^52^. The BMI of these women was first recorded at 12^th^ gestational weeks, which was used as the baseline BMI. Baseline BMI was categorised into underweight (<18.5 kg/m^2^), normal (18.5–24.9 kg/m^2^), overweight (25–29.9 kg/m^2^) and obese (≥30 kg/m^2^) according to the WHO definition. Educational level of the patients was classified into low (elementary school or less), middle (high school) and high (college and above).

Emergency caesarean deliveries included CSs performed after the onset of labour, suspected fetal distress, intrapartum or severe antepartum haemorrhage. Gestational hypertension and preeclampsia were defined according to the American College of Obstetricians and Gynaecologists (ACOG) guideline 2013, the severity of preeclampsia was analysed (with severe features or without severe features)^53^. We also subdivided preeclampsia into different onset types (before or after 34 gestational weeks)^54^. GDM was diagnosed by 75 g oral glucose tolerance test (OGTT) recommended by American Diabetes Association^55^. All patients of ICP were confirmed by associated clinical manifestations and blood biochemical tests^56^. PPH was defined according to the ACOG practice bulletin^57^. Intrauterine growth restriction was defined <10th centile of normal weight in accordant with 2013 ACOG practice bulletin no. 134^58^. A low Apgar score was defined as a score of <7 at 5 minutes.

### Statistical analysis

The department of health statistics of west China school of public health provided the computerized randomization program and statistical support. Data were anonymized and double entered into a customized database. Statistical analysis was performed using SPSS version 19.0 (IBM, Amonki, NY, USA). We compared maternal and perinatal outcomes of different age groups, using women aged 20–29 years as referent group. A two-tailed t test and a χ2 test were used to compare the clinical characteristics between the groups. Independent risk factors were analysed and identified by univariate and multivariate logistic regression analyses, in which possible confounding factors were taken into account. In the investigation of fetal outcomes we excluded pregnancies ending in stillbirth. P < 0.05 was considered statistically significant.

In consideration of the possible different effect of maternal baseline BMI and parity in different age levels, we tested the interaction between maternal baseline BMI with maternal age and parity with maternal age. We found there were no interaction between maternal age and BMI. Primiparity had different influence on selective CS (p value for interaction term p = 0.00), emergency CS (p = 0.01) and GDM (lifestyle intervention) (p = 0.02).

## Electronic supplementary material

Supplementary table S1 and S2
