# Supplementary material for: Pregnancy Outcomes in Women of Advanced Maternal Age: a Retrospective Cohort Study from China
Source: Sci Rep. 2018 Aug 16;8:12239. doi: 10.1038/s41598-018-29889-3 (PMC6095911; doi:10.1038/s41598-018-29889-3)
Supplement: Supplementary file 1 — Supplementary table S1 and S2 [file 41598_2018_29889_MOESM1_ESM.pdf]

Pregnancy Outcomes in Women of Advanced Maternal Age: a Retrospective Cohort Study from China

Dan Shan[1, 4], Pei-Yuan Qiu[2], Yu-Xia Wu[1], Qian Chen[1], Ai-Lin Li[3], Sivakumar Ramadoss[4], Ran-Ran Wang[5] Ya-Yi Hu[1,*]

[1] Department of Gynaecology and Obstetrics, West China Second University Hospital, Sichuan University, Chengdu, China; Key Laboratory of Birth Defects and Related Diseases of Women and Children, Sichuan University, Ministry of Education.

[2] West China School of Public Health, Sichuan University, Chengdu, Sichuan, China.

[3] Department of Ophthalmology, Chengde Medical University, Chengde, Hebei, China.

[4] Department of Obstetrics and Gynaecology and Department of Molecular and Medical Pharmacology David Geffen School of Medicine at University of California at Los Angeles; Jonsson Comprehensive Cancer Centre, Los Angeles, CA.

[5] West China School of Pharmacy, Sichuan University, Chengdu, Sichuan, China.

***Address correspondence to**

Yayi Hu, Ph.D, Department of Obstetrics and Gynaecology, West China Second University Hospital, Sichuan University, Chengdu, Sichuan, China; No. 20, Renmin South Road, Section 3, Chengdu, 610041 China; Tel: +86-28-85501351; +86 15882486697; Fax: +86-28-85502391; E-mail: yayihuscu@sina.com; yayi.hu@163.com

Supplementary Table S1: Crude and adjusted relative risks of the association between maternal baseline BMI, parity and conceiving method with adverse pregnancy outcomes.

	Maternal overweight and obese		Primiparity		ART	
	Crude OR [95% CI]	Adjusted OR[a] [95% CI]	Crude OR [95% CI]	Adjusted OR[a] [95% CI]	Crude OR [95% CI]	Adjusted OR[a] [95% CI]
**Maternal Outcomes**						
**Selective CS**	1.13 [0.90 1.41]	0.95 [0.75 1.23]	0.87 [0.75 1.01]	0.85 [0.70 1.02]	2.39 [1.79 3.18] [b]	1.780 [1.31 2.42] [b]
**Emergency CS**	1.54 [1.20 1.97] [b]	1.57 [1.22 2.02] [b]	1.06 [0.89 1.26]	1.36 [1.00 1.68]	0.74 [0.52 1.06]	0.74 [0.51 1.07]
**ART**	1.07 [0.73 1.58]	1.11 [0.73 1.70]	8.07 [5.39 12.08] [b]	7.20 [4.56 11.38] [b]		
**Preeclampsia**	3.80 [2.56 5.64] [b]	3.15 [2.09 4.73] [b]	0.85 [0.59 1.23]	1.09 [0.69 1.74]	1.42 [0.79 2.57]	0.86 [0.45 1.65]
**Severe preeclampsia**	3.88 [2.42 6.24] [b]	3.47 [2.13 5.67] [b]	0.93 [0.59 1.45]	1.39 [0.80 2.44]	0.93 [0.4 2.17]	0.59 [0.24 1.46]
**Early onset preeclampsia**	3.81 [2.11 6.87] [b]	3.68 [2.01 6.76] [b]	1.13 [0.64 2.00]	1.71 [0.85 3.44]	1.30 [0.51 3.31]	0.83 [0.30 2.26]
**Gestational Hypertension**	2.69 [1.44 5.02] [b]	2.37 [1.25 4.49] [b]	0.75 [0.42 1.32]	0.72 [0.36 1.47]	1.30 [0.51 3.31]	1.02 [0.37 2.80]
**GDM**	1.73 [1.37 2.18] [b]	1.53 [1.20 1.95] [b]	1.01 [0.85 1.20]	1.06 [0.86 1.31]	1.83 [1.38 2.43] [b]	1.27 [0.94 1.73]
**GDM [lifestyle intervention]**	1.41 [1.10 1.83] [b]	1.23 [0.95 1.61]	1.23 [1.02 1.48] [b]	1.29 [1.02 1.62] [b]	1.96 [1.46 2.63] [b]	1.18 [0.86 1.62]
**GDM [on insulin]**	2.21 [1.49 3.26] [b]	2.19 [1.46 3.27] [b]	0.54 [0.39 0.76] [b]	0.58 [0.39 0.87] [b]	1.04 [0.58 1.86]	1.44 [0.76 2.72]
**ICP**	1.09 [0.68 1.75]	1.06 [0.66 1.71]	1.18 [0.86 1.62]	1.33 [0.91 1.95]	0.97 [0.54 1.74]	0.89[0.48 1.65]
**Placental abruption**	0.80 [0.28 2.28]	0.81 [0.28 2.36]	0.85 [0.44 1.63]	0.81 [0.37 1.76]	0.65 [0.15 2.71]	0.70 [0.16 3.11]
**Placenta Praevia and Vasa Praevia**	1.36 [0.96 1.92]	1.15 [0.81 1.65]	0.55 [0.43 0.71] [b]	1.26 [1.16 1.36] [b]	1.23 [0.81 1.87]	1.50 [0.95 2.38]
**PPH**	1.42 [0.89 2.28]	1.27 [0.78 2.05]	0.44 [0.30 0.64] [b]	0.63 [0.40 0.98] [b]	0.91 [0.47 1.77]	1.37 [0.67 2.79]
**Abnormality of fetal presentation [non-cephalic]**	0.78 [0.48 1.28]	0.75 [0.46 1.24]	1.34 [0.99 1.82]	1.63 [1.00 2.36]	1.32 [0.8 2.2]	1.07 [0.62 1.83]
**Fetal outcomes**						
**Preterm birth [<37 weeks]**	1.44 [1.04 1.99] [b]	1.28 [0.91 1.78]	0.50 [0.39 0.63] [b]	0.62 [0.46 0.83] [b]	0.72 [0.45 1.16]	0.97 [0.58 1.61]
**Low birthweight [<2500g]**	1.75 [1.16 2.62] [b]	1.57 [1.04 2.37] [b]	0.63 [0.46 0.86] [b]	0.78 [0.54 1.14]	1.03 [0.60 1.78]	1.27 [0.71 2.30]
**Macrosomia [>4000g]**	1.28 [0.80 2.04]	1.32 [0.82 2.13]	0.81 [0.57 1.34]	0.78 [0.52 1.19]	0.88 [0.46 1.71]	1.02 [0.51 2.05]
**IUGR**	1.02 [0.35 2.97]	0.91 [0.31 2.67]	0.94 [0.46 1.91]	0.90 [0.33 2.51]	1.05 [0.61 1.82]	1.11 [0.31 3.98]
**Apgar score <7 at 5 minute**	0.80 [0.10 6.51]	0.75 [0.09 6.29]	1.20 [0.37 3.95]	3.13 [0.75 13.17]	N	N
**NICU admission**	1.24 [0.80 1.92]	1.11 [0.71 1.74]	0.57 [0.41 0.79] [b]	0.74 [0.50 1.10]	1.31 [0.78 2.2]	1.81 [1.00 3.20]
**Respiratory complications**	1.51 [0.79 2.88]	1.45 [0.75 2.80]	1.34 [0.81 2.22]	1.13[0.62 2.06]	0.98 [0.39 2.47]	1.04 [0.39 2.77]

Note:

CI: confidence interval; OR = odds ratio; ART: assisted reproductive techniques; GDM: gestational diabetes mellitus;

ICP: Intrahepatic cholestasis of pregnancy; PPH: postpartum haemorrhage; IUGR: Intrauterine growth restriction;

NICU: neonatal intensive care unit.

[a] Adjusted for gravidity parity maternal baseline BMI conceiving method insurance education and residence. In the comparison of “conceived by ART”, the aforementioned confounding factors were considered except for conceiving method. [b] Denotes significance with a CI that does not cross 1. N denotes no infants form ART pregnancies had low apgar score at 5 minute.

In comparison of the influence of maternal baseline BMI, patients of normal BMI were used as the reference group. Only the risks of patients of overweight and obese group was reported, for most of the ORs in patients of lower BMI had no statistical significance. In comparison of the influence of parity, multiparous women were used as the reference group. In comparison of the influence of conceiving method, women of spontaneous pregnancies were used as the reference group. In the analysis of fetal outcomes, 2796 patients were analysed excluding pregnancies ending in stillbirth.

Supplementary Table S2： Crude and adjusted relative risks of the association between maternal educational level, residence and insurance with adverse pregnancy outcome.

	**Educational level: low**		**Residence: rural**		**Insurance**	
	**Crude OR [95% CI]**	**Adjusted OR[a] [95% CI]**	**Crude OR [95% CI]**	**Adjusted OR[a] [95% CI]**	**Crude OR [95% CI]**	**Adjusted OR[a] [95% CI]**
**Maternal Outcomes**						
**Selective CS**	1.33 [1.01 1.75] [b]	1.07 [0.80 1.44]	0.71 [0.57 0.87] [b]	0.77 [0.61 0.98] [b]	1.05 [0.91 1.23]	1.03 [0.88 1.22]
**Emergency CS**	1.08 [0.78 1.49]	1.07 [0.77 1.50]	1.13 [0.89 1.45]	1.12 [0.86 1.46]	0.98 [0.81 1.18]	0.98 [0.82 1.18]
**ART**	1.2 [0.68 2.13]	1.01 [0.54 1.89]	0.54 [0.34 0.84] [b]	1.39 [0.84 2.32]	1.18 [0.89 1.57]	1.01 [0.74 1.38]
**Preeclampsia**	2.92 [1.67 5.11] [b]	2.10 [1.16 3.80] [b]	1.98 [1.28 3.07] [b]	2.10 [1.29 3.43] [b]	0.61 [0.42 0.89] [b]	0.66 [0.45 0.98] [b]
**Severe preeclampsia**	3.27 [1.69 6.30] [b]	2.40 [1.20 4.79] [b]	3.12 [1.87 5.21] [b]	3.68 [2.07 6.54] [b]	0.80 [0.51 1.27]	0.91 [0.56 1.46]
**Early onset preeclampsia**	3.21 [1.36 7.58] [b]	2.51 [1.02 6.19] [b]	2.51 [1.32 4.78] [b]	3.23 [1.57 6.63] [b]	1.46 [0.79 2.69]	1.54 [0.81 2.92]
**Gestational Hypertension**	2.51 [1.15 5.49] [b]	1.82 [0.80 4.12]	1.03 [0.47 2.25] [b]	1.10 [0.48 2.52]	1.01 [0.56 1.80]	1.04 [0.58 1.90]
**GDM**	1.8 [1.34 2.42] [b]	1.48 [1.09 2.02] [b]	0.9 [0.71 1.14]	0.97 [0.74 1.26]	0.80 [0.67 0.95] [b]	0.78 [0.65 0.93] [b]
**GDM [lifestyle intervention]**	1.49 [1.07 2.06] [b]	1.21 [0.86 1.70]	0.85 [0.65 1.11]	0.97 [0.73 1.30]	0.80 [0.66 0.96] [b]	0.78 [0.64 0.95] [b]
**GDM [on insulin]**	2.24 [1.38 3.64] [b]	1.98 [1.19 3.32] [b]	1.12 [0.71 1.76]	1.02 [0.62 1.66]	0.89 [0.64 1.23]	0.84 [0.60 1.18]
**ICP**	1.07 [0.61 1.89]	0.99 [0.55 1.77]	1.37 [0.89 2.10]	1.42 [0.89 2.27]	0.73 [0.53 1.01]	0.78 [0.56 1.08]
**Placental abruption**	0.94 [0.28 3.19]	0.89 [0.23 3.14]	1.02 [0.42 2.51]	1.11 [0.42 2.91]	2.00 [0.94 4.26]	2.06 [0.95 4.47]
**Placenta Praevia and Vasa Praevia**	2.13 [1.44 3.16] [b]	1.68 [1.11 2.55] [b]	1.53 [1.11 2.11] [b]	1.34 [0.94 1.90]	0.93 [0.72 1.19]	0.97 [0.75 1.27]
**PPH**	2.99 [1.81 4.93] [b]	2.56 [1.51 4.35] [b]	2.01 [1.30 3.12] [b]	1.53 [0.94 2.48]	1.08 [0.75 1.54]	1.13 [0.77 1.64]
**Abnormality of fetal presentation [non-cephalic]**	1.01 [0.56 1.81]	0.98 [0.54 1.78]	0.89 [0.58 1.38]	1.00 [0.63 1.60]	0.83 [0.61 1.13]	0.80 [0.58 1.11]
**Fetal outcomes**						
**Preterm birth [<37 weeks]**	2.59 [1.80 3.73] [b]	2.13 [1.45 3.11] [b]	2.12 [1.58 2.84] [b]	1.47 [1.07 2.04] [b]	0.96 [0.78 1.22]	1.12 [0.87 1.43]
**Low birthweight [<2500g]**	2.78 [1.77 4.35] [b]	2.33 [1.45 3.72] [b]	1.80 [1.23 2.64] [b]	1.37 [0.90 2.09]	0.94 [0.69 1.28]	1.05 [0.76 1.45]
**Macrosomia [>4000g]**	0.58 [0.26 1.26]	0.61 [0.27 1.35]	0.91 [0.55 1.49]	0.87 [0.51 1.49]	0.93 [0.66 1.32]	0.87 [0.60 1.24]
**IUGR**	2.83 [0.86 9.25]	2.43 [0.71 8.33]	1.36 [0.54 3.41]	0.90 [0.32 2.51]	0.77 [0.38 1.57]	0.91 [0.44 1.88]
**Apgar score <7 at 5 minute**	1.05 [0.13 8.75]	0.56 [0.06 5.12]	5.40[1.44 20.18] [b]	9.35 [2.08 42.09] [b]	1.71 [0.45 6.44]	2.29 [0.58 8.97]
**NICU admission**	2.50 [1.57 3.98] [b]	1.90 [1.17 3.09] [b]	2.59 [1.78 3.77] [b]	2.02 [1.34 3.05] [b]	1.01 [0.73 1.38]	1.19 [0.85 1.66]
**Respiratory complications**	1.30 [0.57 2.97] [b]	1.03 [0.44 2.45]	0.94 [0.45 1.94]	1.16 [0.53 2.50]	1.9 [1.07 3.37] [b]	1.84 [1.03 3.33] [b]

Note:

CI: confidence interval; OR = odds ratio; ART: assisted reproductive techniques; GDM: gestational diabetes mellitus; ICP: Intrahepatic cholestasis of pregnancy; PPH: postpartum haemorrhage; IUGR: Intrauterine growth restriction; NICU: neonatal intensive care unit.

[a] Adjusted for gravidity parity maternal baseline BMI conceiving method insurance education and residence. In the comparison of "conceived by ART", the aforementioned confounding factors were considered except for conceiving method. [b] Denotes significance with a CI that does not cross 1.

In comparison of the influence of education, patients of high educational level were used as the reference group.

Only the risks of the low educational level group was reported, for most of the ORs of patients in middle educational level group had no statistical significance. In comparison of the influence of residence, patients living in metropolitan were used as the reference group. Only the risks of living in rural area was reported, for most of the ORs of patients living in remote area had no statistical significance. In comparison of the influence of insurance, patients who had no insurance was used as the reference group. In the analysis of fetal outcomes,2796 patients were analysed excluding pregnancies ending in stillbirth.
